# Serum levels of hormones regulating appetite in patients with cystic fibrosis − a single-center, cross-sectional study

**DOI:** 10.3389/fendo.2022.992667

**Published:** 2022-10-13

**Authors:** Sabina Galiniak, Rafał Podgórski, Marta Rachel, Artur Mazur

**Affiliations:** Institute of Medical Sciences, Medical College, Rzeszów University, Rzeszow, Poland

**Keywords:** appetite, cystic fibrosis, endocrine system, hormones, ghrelin

## Abstract

Cystic fibrosis (CF), which is the most common inherited genetically determined disease caused by a mutation in the gene for the CF transmembrane conductance regulator protein. Pulmonary failure is the leading cause of death in this population, while the dysregulation of endocrine system creates significant disorders, including malnutrition, underweight, and CF-related diabetes. Therefore, the objective of our study was to determine the following hormones in the serum of patients with CF: ghrelin, putative peptide YY (PYY), Agouti-signaling protein (ASP), and alpha-melanocyte-stimulating hormone (α-MSH). To our knowledge, serum levels of PYY, ASP, and α-MSH have not yet been assessed in CF. For this purpose, we measured hormone levels using enzyme-linked immunosorbent assays in 38 patients from the local CF care center, as well as 16 sex- and age-matched healthy controls. Moreover, we estimated the correlations between the tested hormones and the parameters of the patients’ clinical status. In this study, we found sinificantly reduced serum levels of ghrelin and ASP in patients with CF (p<0.01). There was no difference in PYY and α-MSH levels between participants with CF and healthy subjects. Furthermore, there was no difference in hormone levels between females and males with CF. The type of gene mutation (homozygous or heterozygous for ΔF508) had no effect on hormone levels. Ghrelin was negatively correlated with age, body mass index, and C-reactive protein. PYY was negatively associated with the age of the patients. Hormone dysregulation in CF may contribute to decreased appetite, as well as many other disturbed processes. Therefore, ghrelin appears to play a key role in the regulation of energy management of CF. Future multicenter and multidisciplinary studies should focus on an unequivocal understanding of the role of these hormones in CF.

## Introduction

Cystic fibrosis (CF) is a multisystem disease caused by mutations in a single gene located on human chromosome 7 that encodes the cystic fibrosis transmembrane conductance regulator (CFTR) protein − an epithelial chloride channel with wide tissue expression. CF affects many organs, including the lungs, exocrine and endocrine pancreas, liver and intestines, bone, sweat gland, and male reproductive tract (1). Nevertheless, with greater awareness of the management of CF and access to modern treatment, patients with CF live longer (2, 3). Consequently, patients with CF develop comorbidities, including endocrine disorders. Endocrine dysregulation in CF includes all the following: pubertal delay, infertility, CF-related diabetes, underweight, metabolic bone disease, and failure to thrive (4–6). The greatest interest of researchers is the study of insulin disorders and the development of various forms of diabetes (7, 8). Notwithstanding, other hormonal disorders affect the population of patients with CF, including hormones that regulate appetite and the reproductive system (9–12).

Therefore, the objective of our study was to determine the following hormones in the serum of CF patients: ghrelin, putative peptide YY (PYY), Agouti-signaling protein (ASP), and alpha-melanocyte-stimulating hormone (α-MSH). Ghrelin is a multifaceted gut hormone and plays a crucial role in the release of growth hormones, food intake, fat deposition, glucose and energy homeostasis, cardioprotection, muscle atrophy, and bone metabolism (13). PYY is released from cells in the ileum and colon in response to food intake and inhibits hunger, gastric motility, and increases water and electrolyte absorption in the colon (14, 15). Subsequently, ASP and α-MSH are responsible for the pigmentation mainly of hair and skin; however, they are also involved in feeding behavior, energy homeostasis, sexual activity, and body fat deposition (16–19). To our knowledge, serum levels of PYY, ASP, and α-MSH in CF patients have not yet been determined. We also assessed whether the hormone level was related to the sex of the patients and the type of CFTR mutation. Moreover, we tried to assess the correlations between the tested hormones and the parameters of the patients’ clinical status.

## Material and methods

### Ethical issues

The Rzeszów University Bioethics Committee approved the study protocol (2022/023). All procedures performed in studies involving human participants were in accordance with the ethical standards of the institutional and/or national research committee and with the Declaration of Helsinki of 1964 and its subsequent amendments or comparable ethical standards. The informed consent of the patient and/or legal guardian was obtained in writing.

### Study group

A cross-sectional study of a single center was carried out on thirty-eight patients with CF and sixteen control patients. Participants were recruited from the CF clinic of the Department of Allergology and Cystic Fibrosis, Provincial Hospital No. 2 in Rzeszow from February to October 2021.

The study included Caucasian patients with CF and a confirmed diagnosis based on determination of sweat chloride, genetics, and immune-reactive trypsin tests in neonatal age (patients born in or after 2009). Patients met all of the following inclusion criteria to be eligible for participation in this study: forced expiratory volume in the first second (FEV_1_) greater than 35% of predicted stable pulmonary disease as defined by both clinical impressions and no hospitalizations within 1 month of screening. The exclusion criteria were as follows: heart failure and liver insufficiency, psychiatric disorders, lung transplantation, cystic fibrosis-related diabetes, obesity, corticosteroids treatment, gastrostomy tube feeds or parenteral nutrition and appetite stimulants. Furthermore, if the patients were unable to perform spirometry and refused to participate in the study, they were excluded. All patients with CF suffered from pancreatic insufficiency and received regularly pancreatic enzyme replacement therapy (Creon 25000, Solvay Pharmaceutical Inc., Marietta, Georgia, USA). Patients were also treated with human DNase I recombinant (Pulmozyme, Genentech Inc., San Francisco, California, USA; one 2.5 mg ampoule inhaled once daily using a nebulizer), fat-soluble vitamins in the form of ADEK tablets (Scandipharm, Birmingham, Alabama, USA), nutrition drinks (Nutrison Protein Plus, Nutricia, Poland) and inhalation of 3–10% sodium chloride 3–4 times daily. All patients were clinically evaluated, showing no signs or symptoms of malabsorption, and had a stable weight for at least 2 months prior to the study. Information on the type of CFTR mutation (homozygous or heterozygous for ΔF508) as well as other clinical parameters was obtained from hospital patient records. Additionally, information on bacterial infections with *Pseudomonas aeruginosa* and/or *Staphylococcus aureus* in the sputum was obtained from hospital patient records.

Healthy patients aged 10–38 were recruited at the same time from the local clinic. The control group consisted of volunteers who had no diseases in medical history or physical examination. Healthy participants did not receive any treatment, including supplements, 30 days before the study. All participants in the control group had normal pulmonary function tests. In addition, all participants had anthropometric measurements. BMI was calculated as kg/m^2^ for adult participants, while z-score was calculated for pediatric patients and controls.

### Spirometry

All participants performed spirometry with a standard spirometry device (Lungtest 1000, MES, Kraków, Poland) according to recommendations (20). We calculated the mean value of the last half year for FEV_1_ expressed as a percentage of the predicted value for age and sex.

### Blood sampling

Blood samples were collected between 8 am and 10 am after a night of fasting and placed in blood collection tubes. The collected serum was incubated at room temperature for at least 30 min, and centrifuged (1500×g, 10 min, 4°C). Subsequently, the serum was transferred to cryovials and placed immediately in the freezer at −80°C until further analysis. The serum sample was thawed only once on the day of analysis.

### Blood counts and serum analysis

Blood morphology was performed using a hematology analyzer (Siemens Healthineers, Germany). C-reactive protein (CRP) concentration was estimated using the dry chemistry immunological method on a VITROS 250 analyzer (Ortho Clinical Diagnostics, Johnson and Johnson, USA).

### Concentration of ghrelin, PYY, ASP, and α-MSH in serum

Serum hormone concentrations after an overnight fast were measured in duplicates with a previous dilution using commercially available enzyme-linked immunosorbent assays (Wuhan Fine Biotech Co., Ltd., Wuhan, China), according to the manufacturer’s protocol.

### Statistical analysis

All statistical analyses were performed with the STATISTICA software package (version 13.3, StatSoft Inc. 2017, Tulsa, OK, USA). Data are expressed as mean and SD, as well as range. The normality of the distribution was validated using the Shapiro-Wilk test, as well as skewness values. The Mann-Whitney U test was used to compare differences between two independent groups. The correlation analysis was performed using the Spearman correlation test, assuming linear dependence with α=0.05.

## Results

In our study, seventeen females and eleven males with CF were included in the CF group, as well as healthy ten females and six males were recruited into the control group. Basic characteristics, clinical laboratory values, and lung function indices for patients with CF and healthy participants are shown in **Table 1**.

**Table 1:** Basic characteristics of study participants*.

				CF	Healthy controls	*p*
Sex (F/M)				17/21	10/6	* *
Age (years)	mean ± SD			19.58 ± 7.9	19.25 ± 7.3	0.855
	range			10−39	10−38	
Height (cm)	mean ± SD			157.56 ± 18.1	160.56 ± 15.1	0.598
	range			124−188.5	130−180
Weight (kg)	mean ± SD			50.03 ± 13	58.75 ± 13.9	0.038
	range			22.1−76	34−82
BMI (kg/m^2^), patients >20 years	mean ± SD			21.08 ± 2.87	23.16 ± 2.42	0.068
	range			17.2−25.9	18.7−25.6
BMI (Z-score), patients <20 years	mean ± SD			-0.79 ± 1.13	0.16 ± 0.48	0.433
	range			-2.09−1.07	-0.21−1.01
Genotype						
Homozygous ΔF508, n (%)				30 (78.9)	*−*	−
Heterozygous ΔF508, n (%)				8 (21.1)	*−*	−
Clinical laboratory markers						
WBC (10^3^/µL)	mean ± SD			9.95 ± 3.6	7.46 ± 2.3	0.022
	range			5.1−19.3	4.3−10.5
NEU (%)	mean ± SD			61.01 ± 15.3	59.12 ± 6.1	0.605
	range			25.1−82.3	50.6−68.6
CRP (mg/L)	mean ± SD			5.32 ± 4.8	1.92 ± 1.2	0.001
	range			0.5−22	0.3−4.2
Pulmonary function						
FEV_1_ (% predicted)	mean ± SD			86.35 ± 27	102.4 ± 8.2	0.006
	range			35−142	97−127
Severity of disease						
Mild (FEV_1_>75%), n (%)				23 (60.5)	*−*	*−*
Moderate (FEV_1_>45%, <75%), n (%)				9 (23.7)	*−*	*−*
Severe (FEV_1_<45%), n (%)				6 (15.8)	*−*	*−*
Bacterial infection						
*P. aeruginosa*, n (%)				9 (23.7)	*−*	*−*
*S. aureus*, n (%)				11 (28.9)	*−*	*−*
Co−infected with *P. aeruginosa* and *S. aureus*, n (%)				9 (23.7)	*−*	*−*
Uninfected, n (%)				9 (23.7)	16 (100)	*−*

*Data are presented as mean ± standard deviation; range or count and percentage. BMI, body mass index; WBC, white blood cells; NEU, neutrophils; CRP, C-reactive protein; FEV_1_, forced expiratory volume in 1 second.

The mean age in the CF patient group was 19.58 ± 7.9 years, while the healthy subjects were 19.25 ± 7.3 years old. There were no differences in the age and height of the study groups. CF patients had statistically lower body weight than healthy controls. The BMI was similar in adult patients with CF and healthy volunteers. 30 participants with CF were homozygous for ΔF508 and 8 (21.1%) were heterozygous. CF patients had significantly higher white blood cell counts and CRP levels. Lung function was decreased in the CF group compared to healthy subjects. Based on the spirometry results, more than 60% of the patients had mild disease, 23.7% of the patients had moderate and 15.8% of the patients had severe disease. Among patients with CF, 9 were infected with *P. aeruginosa*, 11 were infected with *S. aureus*, 9 were co-infected with *P. aeruginosa* and *S. aureus*, while 9 were uninfected.

**Figure 1:**
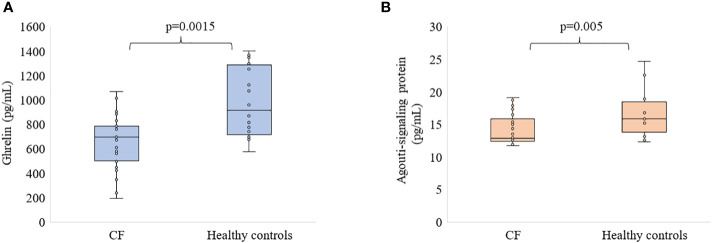
Level of ghrelin **(A)** and ASP **(B)** in patients with CF as compared to healthy participants.

**Figure 2:**
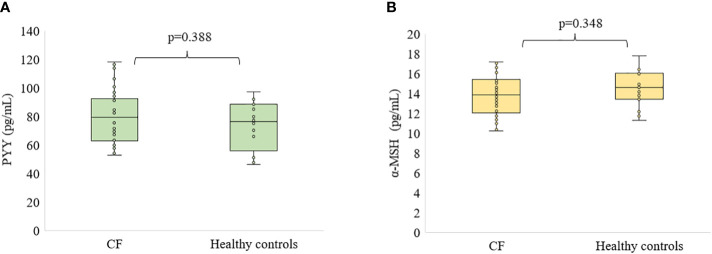
Level of PYY **(A)** and α-MSH **(B)** in patients with CF as compared to healthy participants.

The levels of hormones studied are presented in **Figures 1**, **2**. The ghrelin level was significantly decreased in the serum of patients with CF compared to healthy subjects (p<0.01, **Figure 1A**). Similarly, ASP concentration was significantly lower in participants with CF than in healthy controls (p<0.01, **Figure 1B**). However, we did not find any differences in the level of PYY and α-MSH (**Figure 2**).

**Table 2** presents a comparison of the concentration of studied hormones in females and males with CF. We did not observe any difference in hormone levels between females and males with CF.

**Table 2:** Hormone levels by sex of CF patients*.

	Females with CF	Males with CF	*p*
Ghrelin (pg/ml)	639.34 ± 235.74	681.56 ± 185.32	0.6322
PYY (pg/ml)	85.24 ± 20.15	76.6 ± 17.64	0.1676
ASP (pg/ml)	13.25 ± 1.54	14.62 ± 2.45	0.1002
α-MSH (pg/ml)	14.17 ± 2.27	13.62 ± 1.9	0.5123

*Data are presented as mean ± standard deviation.

Moreover, there was no association between the type of CFTR mutation and the level of the tested hormones (**Table 3**).

**Table 3:** Hormone levels by CFTR mutation of CF patients*.

	Homozygous ΔF508	Heterozygous ΔF508	*p*
Ghrelin (pg/ml)	660.28 ± 226.26	663.66 ± 150.02	0.9217
PYY (pg/ml)	80.33 ± 20.45	80.96 ± 13.57	0.7069
ASP (pg/ml)	13.78 ± 2.17	14.88 ± 2.12	0.0959
α-MSH (pg/ml)	14.04 ± 2.19	13.26 ± 1.48	0.4064

*Data are presented as mean ± standard deviation.

The next step was to try to determine the correlation between the levels of the hormones studied and the clinical parameters of CF patients. Associations between hormones and clinical data are presented in **Table 4**. In addition, we present the correlations between the tested and other appetite regulating hormones, including leptin, neuropeptide Y, kisspeptin (KISS) and proopiomelanocortin (POMC), which we previously determined by us (data not shown).

**Table 4:** Spearman’s rank correlation coefficients and p values*.

		Age	BMI (kg/m^2^) >20 years	BMI (z-score), <20 years	CRP	FEV_1_	Leptin	Neuropeptide Y	KISS	POMC	Ghrelin	ASP	PYY
**Ghrelin**	**R**	-0.555	-0.8964	0.3461	-0.401	0.2564	-0.7323	0.1134	0.5375	0.386			
	**p**	0.0054	0.0001	0.2466	0.0169	0.137	0.0001	0.5164	0.0009	0.0219			
**ASP**	**R**	-0.0135	0.111	0.1676	0.1028	-0.086	-0.0808	0.048	-0.0546	-0.1671	0.0249		
	**p**	0.9359	0.6523	0.5348	0.5389	0.6068	0.6495	0.774	0.7447	0.316	0.887		
**PYY**	**R**	-0.3707	-0.3747	-0.1617	-0.2762	0.1746	-0.4001	-0.047	-0.0158	0.2888	0.3098	-0.0158	
	**p**	0.0219	0.114	0.8202	0.0931	0.2943	0.019	0.7791	0.9246	0.079	0.0701	0.9246	
**α-MSH**	**R**	-0.2786	-0.2921	0.2647	-0.0549	0.0811	-0.0411	-0.4255	-0.0403	-0.1676	0.1388	-0.0561	0.2411
	**p**	0.0949	0.2396	0.3218	0.7467	0.633	0.8203	0.0086	0.8127	0.3214	0.4334	0.7411	0.1505

*BMI, body mass index; CRP, C-reactive protein; FEV_1_, forced expiratory volume in 1 second; KISS, kisspeptin; POMC, proopiomelanocortin.

Ghrelin was negatively correlated with age, BMI in patients older than 20 years, and CRP. Furthermore, we found a strong negative correlation between ghrelin and leptin concentration in the sera of patients with CF (R=-0.7323, p<0.001). Ghrelin was also positively correlated with the level of KISS (R=0.5375, p<0.001) and POMC (R=0.386, p<0.05). PYY was negatively associated with the age of the patients (R=-0.3707, p<0.05) as well as with serum leptin levels (R=-0.4001, p<0.05). α-MSH negatively correlated with the level of neuropeptide Y. ASP levels were not correlated with any of the parameters tested and their concentration was not correlated with other hormones studied. It should be noted that none of the hormones studied was correlated with the results of spirometry.

## Discussion

Our study presents for the first time circulating levels of the hormones that regulate energy metabolism and nutrition, such as the putative peptide YY, the Agouti-signaling protein and the alpha-melanocyte-stimulating hormone in patients with CF. The main findings of our study are a significantly decreased level of ghrelin and ASP in the sera of patients with CF compared to healthy controls. Furthermore, we found that PYY and α-MSH did not differ in CF patients and healthy participants.

Eating and satiation feelings involve complex interactions between many hormones from the gastrointestinal tract to the hypothalamus and subsequent feedback (21). Chronic loss of appetite and malnutrition in CF belong to the most common complication in adolescents and adults with CF, both affecting patients, their families, and physicians (22–24). Currently, appetite stimulants are used to obtain optimal BMI, nutritional status, and consequently improve lung function in CF (25, 26). Nonetheless, many of them may have an adverse effect on the clinical status of patients (27, 28). Malnutrition results from a discrepancy between energy/nutrient requirements and food intake, which can be caused by malabsorption (29). Furthermore, several reports also point to disturbances in hormones that regulate appetite (10, 30).

We found a decreased level of ghrelin in CF participants. Contrary to our results, the levels of ghrelin in the serum of children with CF patients were significantly higher than those of the control group (31). On the other hand, the plasma ghrelin level did not differ between healthy controls and patients with mild or moderate disease in a study conducted among adults with CF (10). However, a lower level of ghrelin compared to healthy controls was also reported, which is consistent with our results (11). This indicates a discrepancy that may be influenced by more factors than the disease itself. Similarly to our results, no differences were shown between serum ghrelin concentration levels in relation to sex and the type of CFTR mutation in previous studies (10, 31, 32). Nevertheless, one report presented lower fasting ghrelin levels in CF males compared to CF females (p=0.01) (11).

We have also found significantly lowered level of ASP in CF participants. Human ASP is expressed at the highest levels in adipose tissue where it is a competitive antagonist of the α-MSH to bind to receptor (33). So far, the level of ASP in human serum has not been determined. Reduced levels of ASP may indicate that this hormone is involved in the regulation of eating behavior in CF. Furthermore, ASP stimulates insulin release from the pancreas, therefore the decrease in insulin level in CF may be associated not only with pancreatic damage, but also with a decrease in ASP levels in patients with CF-related diabetes (7, 34). The sex of the patients and the type of CFTR mutations did not influence the level of ASP in CF.

Peripheral PYY has been reported to act as a satiety signal, regulating the termination of individual meals, in part by reducing the production of the hunger-stimulating peptide ghrelin. In our study, we did not find differences in PYY levels between patients with CF and healthy controls. Higher total plasma PYY levels were reported in patients with anorexia nervosa compared to lean, obese, or morbidly obese subjects (15). However, fasting PYY levels were comparable in anorexia nervosa, bulimia nervosa, and in healthy controls in study by Sedlackova et al. (35). Elevated PYY levels have also been reported in critically ill patients, particularly in patients with food intolerance (36). The PYY levels were 25.49 ± 9.79 pg/mL among adolescents with anorexia nervosa and 18.46 ± 9.81 pg/mL in healthy adolescents, which is a much lower concentration than in our study, but this difference may occur due to technical differences in the tests used for the analysis (37). There were no differences in PYY levels between females and males with CF, as well as homozygous and heterozygous for ΔF508. Comparison of PYY levels between male and female rats showed the existence of sex-related differences in early postnatal life, but not in the pubertal or adult stage (38).

The α-MSH, an endogenous neuropeptide derived from POMC, is widely expressed in various tissues and organs and plays an important role in a variety of biological processes, such as energy metabolism, body weight regulation, sexual activity, and exocrine secretion (39). This hormone also has broad anti-inflammatory effects (40). It was shown that α-MSH inhibited leukocyte migration to the lungs in lypopolysaccharide-induced acute lung injury in rats (41). We did not find any difference in the concentration of α-MSH between CF patients and healthy people. Nevertheless, reduced serum α-MSH concentrations were found in patients with osteonecrosis of the femoral head and people with craniocerebral injury compared to healthy controls (42, 43). An elevated serum level of α-MSH was observed in patients with chronic fatigue syndrome and hypothalamic obesity associated with craniopharyngioma (44, 45). The levels of α-MSH were independent of sex and type of CFTR mutations. Taking into account sex, levels of α-MSH were higher in men than in women (10.1 ± 4.3 vs 7.6 ± 3.4 pmol/L, p=0.019) only in obese patients (46). Similarly to our results, plasma levels of a-MSH did not differ significantly between groups in both male and female populations, as previously reported (47). However, in obese men, plasma α-MSH concentrations increased significantly compared to non-obese men (48).

We had also evaluated the hormone correlation between the levels studied and the clinical parameters of the CF patients. To our knowledge, there have been no studies investigating the correlation between the level of PYY, ASP, and α-MSH, and clinical data. We found no correlation between ghrelin level and age of patients. Regarding ghrelin, no age-dependence was reported in a study of children with CF (32). Nevertheless, only children with an average age of 4.5 years were included in this report, which may suggest that such a correlation did not exist (32). Similarly to our study, the ghrelin level was negatively correlated with BMI among adults with CF (R=-0.35, p<0.001) (10). The average concentrations of ghrelin in children with inadequate body weight (low BMI) were higher, but not statistically, than in the subgroup with the normal level of nutrition, which is in line with our results (32). Furthermore, a study on lymphocytes showed that the expression of the ghrelin receptor in the CF group with normal BMI was similar to that of controls; however, it decreased during an acute exacerbation associated with weight loss and returned to baseline after treatment and recovery of weight loss (49). In our study, the level of ghrelin was negatively correlated with CRP. In the presence of a probable infection and worsening of the inflammatory state, ghrelin levels may be affected. Lung function alone may not affect hormone levels, but certainly pro-inflammatory products during exacerbation periods may play a role in this (50). Our results did not show a correlation between ghrelin level and spirometry results, which may indicate that lung function does not affect hormone levels. A strong negative correlation between FEV_1_ and ghrelin (R=-0.62, p<0.001) had been reported in the study by Cohen et al. (10). Furthermore, no linear correlation was observed between leptin and ghrelin values, which is in contradiction to our results (11). The opposite metabolic function of leptin and ghrelin explains the negative correlation between them. The leptin/ghrelin system influences the hypothalamus and transmits peripheral information about the nutritional status of the body and its energy reserves. Stimulation of leptin receptors inhibits the secretion of orexigenic neurotransmitters in the arcuate nucleus of the hypothalamus, such as the neuropeptide Y and Agouti-related protein, and stimulates POMC secretion (51). POMC is degraded by enzyme into α-MSH, which, acting through MC4R receptors located in the paraventricular nucleus, regulates food intake and autonomic nervous system and leads to weight loss (52).

We also found no correlation between ASP, the parameters studied, and other hormones. Furthermore, so far no correlation between ASP and patient clinical data has been described in the literature.

In our study, we showed a negative correlation between the level of PYY and the age of the study participants. PYY levels were influenced by age, thyroid hormones, and growth hormones in rats (38). PYY was not correlated with other clinical parameters of the patients, which is consistent with the report described previously (53). PYY levels appear to be affected by acute exercise, macronutrient composition, adiposity, race, and the composition of fatty acids in dietary fat (54). Moreover, PYY levels were significantly associated with age and cardiovascular risk factors, including hypertension, diabetes, and kidney function, in addition to biomarkers of heart failure and inflammation in patients with acute myocardial infarction (55). However, PYY was negatively correlated with leptin. In our study, there was no correlation between ghrelin levels and PYY, but the result of the correlation analysis was close to statistical significance (p=0.070). PYY is known to inhibit ghrelin expression, and this action occurs at the level of the vagal afferent fibers, the ganglia, and the hypothalamus, especially the arcuate nucleus (56).

In our study, levels of α-MSH were not correlated with age, BMI, CRP, and FEV_1_. Plasma levels of α-MSH were positively correlated with BMI (R=0.560, p<0.05) in obese men (41). On the other hand, negative correlations between α-MSH and BMI levels had previously been demonstrated (57). No correlations were found between plasma α-MSH concentrations and BMI, waist circumference, blood pressure, and heart rate in the total study population (47). α-MSH hormone levels did not correlate significantly with any adiposity or diet composition in the study by Donahoo et al. (46). Nevertheless, α-MSH and the neuropeptide Y were negatively correlated. Data suggest that the α-MSH and neuropeptide Y system interact to control food intake, but the detailed role of this crosstalk in the regulation of energy balance remains unclear (58).

Our study describes the level of hormones in CF that, except ghrelin, have not yet been described in this condition. Additionally, a strong point of our work is the inclusion of adult patients with CF in the study, as much of the research focuses on children and adolescents with CF. Adult patients, which make up an increasing population, are often overlooked. Among the limitations of the study, it could be distinguished that only patients from one health center were included in the study. Furthermore, we did not test for glucose and insulin levels that could significantly improve the study. In addition, children and adolescents have different body composition and sexual development compared to adults, which may affect the analysis of the hormones evaluated in the present study. We also did not analyze the intensity of exercise in the study participants, and it is known to alter ghrelin and PYY levels. Many factors such as age, puberty, race, and body composition all appear to influence appetite-related gut peptides, and therefore should be taken into account when interpreting and designing further studies in children and adolescent populations.

In summary, we found lower serum levels of ghrelin and ASP in serum from CF patients with CF compared to healthy subjects. Moreover, there was no difference in the concentration of PYY and α-MSH. Additionally, sex and type of mutation did not affect hormone levels. In our study, we described some interesting correlations between the level of hormones and the parameters that describe the clinical status of patients. It should be emphasized that none of the hormones was correlated with the spirometry results, indicating that the severity of the disease does not affect the level of hormones. Hormone dysregulation in CF can contribute to decreased appetite as well as many other disturbed processes. Overall, ghrelin appears to play a key role in regulating energy management in CF. Future multicenter and multidisciplinary studies should focus on an unequivocal understanding of the role of these hormones in CF.

## Data availability statement

The original contributions presented in the study are included in the article. Further inquiries can be directed to the corresponding author.

## Ethics statement

The studies involving human participants were reviewed and approved by the Bioethics Committee of Rzeszów University (2022/023). Written informed consent to participate in this study was provided by the participants’ legal guardian/next of kin.

## Author contributions

Conceptualization, SG and AM. Data curation, SG and RP. Formal analysis, SG. Investigation, SG and RP. Methodology, SG and RP. Project administration, SG. Resources, SG and MR. Software, SG. Supervision, SG. Writing−original draft, SG. Writing−review and editing, SG, RP, MR and AM. All authors contributed to the article and approved the submitted version.

## Conflict of interest

The authors declare that the research was conducted in the absence of any commercial or financial relationships that could be construed as a potential conflict of interest.

## Publisher’s note

All claims expressed in this article are solely those of the authors and do not necessarily represent those of their affiliated organizations, or those of the publisher, the editors and the reviewers. Any product that may be evaluated in this article, or claim that may be made by its manufacturer, is not guaranteed or endorsed by the publisher.
